# Adverse effects of ayahuasca: Results from the Global Ayahuasca Survey

**DOI:** 10.1371/journal.pgph.0000438

**Published:** 2022-11-16

**Authors:** José Carlos Bouso, Óscar Andión, Jerome J. Sarris, Milan Scheidegger, Luís Fernando Tófoli, Emérita Sátiro Opaleye, Violeta Schubert, Daniel Perkins

**Affiliations:** 1 International Center for Ethnobotanical Education, Research and Services (ICEERS), Barcelona, Spain; 2 Medical Anthropology Research Center, Department of Anthropology, Philosophy and Social Work, University of Rovira I Virgili, Tarragona, Spain; 3 Department of Neurosciences and Behavior, Ribeirão Preto Medical School, University of São Paulo, São Paulo, Brazil; 4 Research Sherpas, Mieres (Olot), Sabadell, Spain; 5 Psychae Institute, Melbourne, Australia; 6 NICM Health Research Institute, Western Sydney University, Westmead, Australia; 7 Florey Institute of Neuroscience and Mental Health, The Melbourne Clinic Professorial Unit, Department of Psychiatry, University of Melbourne, Melbourne, Australia; 8 Department of Psychiatry, Psychotherapy, and Psychosomatics, University Hospital of Psychiatry Zurich, University of Zurich, Zurich, Switzerland; 9 Interdisciplinary Cooperation for Ayahuasca Research and Outreach (ICARO), School of Medical Sciences, University of Campinas, Campinas, Brazil; 10 Department of Psychobiology, Universidade Federal de São Paulo, São Paulo, Brazil; 11 School of Social and Political Science, University of Melbourne, Melbourne, Australia; 12 School of Population and Global Health, University of Melbourne, Melbourne, Australia; 13 Centre for Mental Health, Swinburne University, Melbourne, Australia; PLOS: Public Library of Science, UNITED STATES

## Abstract

**Introduction:**

Ayahuasca is a plant-based decoction native to Amazonia, where it has a long history of use in traditional medicine. Contemporary ritual use of ayahuasca has been expanding throughout the world for mental health purposes, and for spiritual and personal growth. Although researchers have been conducting clinical trials and observational studies reporting medical and psychological benefits, most of these do not report ayahuasca’s immediate or medium-term adverse effects, so these are underrepresented in the literature. With the expansion of ayahuasca ceremonies from their traditional contexts to countries around the world, there is an important public health question regarding the risk/benefit balance of its use.

**Methods:**

We used data from an online Global Ayahuasca Survey (n = 10,836) collected between 2017 and 2019 involving participants from more than 50 countries. Principal component analysis was performed to assess group effects. Logistic regression analysis was performed to test for adverse effects associated with history of ayahuasca use, clinical, context of use and spiritual effect variables.

**Results:**

Acute physical health adverse effects (primarily vomiting) were reported by 69.9% of the sample, with 2.3% reporting the need for subsequent medical attention. Adverse mental health effects in the weeks or months following consumption were reported by 55.9% of the sample, however, around 88% considered such mental health effects as part of a positive process of growth or integration. Around 12% sought professional support for these effects. Physical adverse effects were related to older age at initial use of ayahuasca, having a physical health condition, higher lifetime and last year ayahuasca use, having a previous substance use disorder diagnosis, and taking ayahuasca in a non-supervised context. Mental health adverse effects were positively associated with anxiety disorders; physical health conditions; and the strength of the acute spiritual experience; and negatively associated with consumption in religious settings.

**Conclusions:**

While there is a high rate of adverse physical effects and challenging psychological effects from using ayahuasca, they are not generally severe, and most ayahuasca ceremony attendees continue to attend ceremonies, suggesting they perceive the benefits as outweighing any adverse effects. Knowing what variables might predict eventual adverse effects may serve in screening of, or providing additional support for, vulnerable subjects. Improved understanding of the ayahuasca risk/benefit balance can also assist policy makers in decisions regarding potential regulation and public health responses.

## Introduction

Ayahuasca is a decoction native to Amazonia, where it has a long history of use in traditional medicine, with samples of its alkaloids having been identified at archaeological sites dating back about 1000 years [1]. Ayahuasca is the Quechua name for both the vine *Banisteriopsis caapi* and the decoctions/admixtures made from it, which contain the beta-carboline, monoamine oxidase inhibitor (MAOI) compounds harmine, harmaline, and tetrahydroharmine (THH). In traditional Amazonian medical systems, from the base of *B*. *caapi* every native healer uses an admixture of local plants, depending on the medical conditions that are intended to be treated. The MAOI properties of *B*. *caapi* increase the bioactivity of many phytochemical compounds, including monoamines especially, by inhibiting their breakdown in the gastrointestinal tract. The most common recipe for ayahuasca, where the MAOI compounds support the bioactivity of a monoamine, involves mixing it with the leaves of the bush *Psychotria viridis* or, depending on the area, with the leaves of the vine *Dyploteris cabrerana*, both containing N,N-Dymethyltriptamine (DMT), a compound that is considered a hallucinogen in its purified form. DMT is included in the Schedule 1 list of the 1971 UN treaty of Psychotropic Drugs. Although DMT is scheduled in the 1971 treaty, the DMT containing plants used in ayahuasca, nor decoctions made from this plant material, are scheduled in international treaties [2]. However, some countries do have local laws prohibiting DMT and/or the plants used in the production of ayahuasca (including *B*. *caapi*).

Despite its ambiguous legal status, in the last decades ayahuasca use has increased internationally [3, 4]. However, contrary to other substances exported from the Americas, like coca, *Psilocybe* mushrooms, *Salvia divinorum*, and tobacco, instead of being used in a denaturalized and deculturated manner, contemporary use typically resembles traditional use in several aspects: 1) the user does not self-administer the brew, but it is administered by a facilitator who has been trained by other experts, generally belonging to some Amazonian medical system tradition or a Brazilian syncretic religious context; 2) ayahuasca’s active principles are not extracted for use in isolation or in synthetic forms; 3) generally it is used in group settings [5]. These factors potentially offer some degree of protection against adverse effects.

Although ayahuasca is considered a hallucinogen, traditional cultures have used it throughout history not to escape from reality, but to better adapt to it [6, 7]. Since health is not an individual experience for traditional cultures, but rather a consequence of alignment between the individual, the community, the natural ecosystem, and the geographic territory, ayahuasca is actually a tool for achieving such an alignment [8]. Furthermore, ayahuasca and other psychoactive ethnobotanicals, like peyote (Lophophora williamsii) and iboga (Tabernanthe iboga), play an important role in traditional rituals as social enhancements, a crucial aspect for preserving mental health [9]. Both the alignment process and the strengthening of social ties are aspects that allow non-native users, when they attend ayahuasca ceremonies, to find better health as a result [10, 11]. Biomedical scientists are now interested in finding clinical applications for ayahuasca, having found improvements in clinical and non-clinical conditions like affective disorders [12–16], drug dependence [17, 18], emotional regulation in personality disorders [19], grief [20, 21], mindfulness and psychological flexibility capabilities [22–27], creativity [28, 29], neuropsychological performance [30], human neuroplasticity [31], and some other psychological and physical diseases of contemporary civilization [32].

The rise of attention on ayahuasca, alongside the revival of scientific interest in psychedelic substances and their therapeutic potential, has several dimensions and important implications in terms of public global health and policy that need to be considered [33]. On one side, ayahuasca is a traditional medicine in innumerable Amazonian cultures that is increasingly medicalized, globalised, commercialised and enfolded into powerful Western demand, and consumerism. Conversely, many Westerners are travelling to traditional locations seeking the supposed healing properties of ayahuasca ceremonies and of perceived powerful curanderos. Some traditional healers are also travelling internationally, sometimes experiencing prosecution due to DMT being a scheduled substance.

The, growing interest in ayahuasca also points to weaknesses in Western medicine, therapeutic and healing regimes. Most people that attend traditional ayahuasca healing ceremonies are seeking healing for mental health and emotional difficulties [5, 23, 34]. Indeed, some consider that traditional ayahuasca medicine may be a tool to combat the current global mental health crisis. Such consideration by international policy may be described as reciprocal Global Mental Health [8], where not only biological psychiatry is exported to traditional societies, but traditional medicine may be exported in decolonial and non-extractive ways to Western societies. Thus, in this complex and polyhedric context and multiple levels of understanding of the expanding ayahuasca phenomenon, it is critical to better understand the balance of risk and benefits related to ceremony participation.

With the globalization of ayahuasca there are now a wide range of different settings in which people can attend ayahuasca ceremonies. These include traditional ceremonies located in the Amazon region, conducted with local practitioners (*curanderos*, *maestros*, *médicos tradicionales* -traditional healers, *taitas*, etc.), or by traditional healers who travel to western countries to deliver ayahuasca ceremonies. The religious context involved ayahuasca consumption in the Brazilian based ayahuasca churches, which are now present in many countries around the world. Finally, there are people in Western settings delivering ayahuasca ceremonies that in some way try to emulate traditional methods, some of whom may have spent time in the Amazon learning the traditional methods. A previous study from our group using data from the Global Ayahuasca Project showed little difference in therapeutic mental health outcomes across these different contexts of use [5]. However, this may not be the case for adverse events, so we believe is an important variable to include.

Ayahuasca’s safety has been demonstrated in clinical trials involving both different dosages and multiple administrations [13, 14, 35–45], as well as in naturalistic settings [21, 24–30]. The main physical side effects are nausea, vomiting, and diarrhoea, but in traditional settings those effects are considered part of the ayahuasca healing process (the subjective experience of perceived ‘spiritual cleansing’) rather than side effects per se [46]. Furthermore, even some psychotomimetic effects are specifically being sought out when people attend ayahuasca ceremonies. Both in traditional and modern settings, for example since visions are considered a source of knowledge [36]. Other common physical side effects reported include changes in body perception and alterations like an electric/tingling feeling, change in body temperature, and dizziness, among others. All adverse physical effects are temporary and are not considered severe [47, 48].

Although there is consensus regarding the general safety of ayahuasca [49], it may induce eventual adverse effects that are likely underrepresented in the literature for a number of reasons: 1) in the clinical trials, subjects are carefully selected, decreasing the probability of their experiencing serious negative effects; 2) most naturalistic studies have relatively small sample sizes; 3) researchers tend to focus on positive effects rather than investigating negative effects; 4) most adverse psychological effects are actually challenging transitory experiences that have no further negative consequences and might result in positive outcomes [50]; 5) it is not always possible to establish causal relationships because side effects are not reported on site [51]; and 6) the actual rate of severe side effects is relatively low [52]. The following reports of adverse effects have been identified in the literature:

- The American Association of Poison Controls Centers’ (AAPCC) National Poison Data System (NPDS) collected 538 calls related to exposure to ayahuasca botanical products between 2005 and 2015. Forty-one cases (7%) reported major clinical manifestations and 296 cases moderate ones (55%). The most common clinical effects were hallucinations (190, 35%), agitation (181, 34%), tachycardia (180, 34%), confusion (99, 18%), hypertension (87, 16%), mydriasis (72, 13%), and vomiting (32, 6%). The most severe effects were seizures (12, 2%), respiratory arrest (7, 1%), and cardiac arrest (4, 1%). Three fatalities were reported. In this study, patient’s characteristic, previous medical conditions, concomitant medication use, and what subjects actually took were unknown [51].
- A systematic review of the published case reports describing psychotic episodes associated with ayahuasca use found three case series and two case reports describing psychotic episodes [53], and at least three other cases have been reported since then [54–56]. Although most of the cases had previous psychiatric diagnoses, a few did not. Some of the cases required antipsychotic medication, but all of them returned to their pre-crisis mental states after a variable amount of time.
- A study involving 32 subjects of a US Santo Daime church also identified side-effects, most commonly nausea (11 subjects), vomiting (9 subjects), and exhaustion in the following days (9 subjects). Other somatic effects, like headache, tachycardia, and muscle spasms, were reported by a few subjects [57]. In a recent retrospective study involving 614 members (regular uses) of the União do Vegetal, a Brazilian religion where ayahuasca is a sacrament, the most common physical effects, as expected, were vomiting (96.74%) and nausea (91.53%). Diarrhoea, shivers, tachycardia, tremor, and tinnitus were other common effects. Surprisingly, persistent physical effects were reported by 41.86% of the participants, among whom the daily lives of 3.89% (10 individuals) were reasonably (n = 9) or very (n = 1) affected in a negative way. Adverse psychological effects (anxiety, disorientation, or distress) were less common; however, persistent adverse psychological effects were reported by 20.68% of the participants (127 individuals), among which 11.81% (n = 15) indicated that their daily lives were moderately (n = 14) or very (n = 1) negatively affected by persistent effects. The total duration of the persistent adverse effects was not recorded. Participants with a reported psychiatric diagnosis experienced adverse effects more frequently than those without [52].
- In a prospective longitudinal study involving 40 subjects who participated in ritual ayahuasca ceremonies [58], 7 referred to an extremely challenging psychological reaction, involving psychotic symptoms in some cases. Among those 7 subjects, 4 had a previous psychiatric diagnosis. The condition of all subjects with psychiatric antecedents improved, and none of those without were found to be worse during the follow-ups [50].
- In a series of controlled clinical trials, while characterizing the pharmacology of ayahuasca with a total of 24 subjects using low (0.5mg/DMT/kg), medium (0.75mg/DMT/kg), and high (1mg/DMT/kg) doses of ayahuasca [40, 41], subjects scored active doses higher than a placebo in terms of the Visual Analogue Scales “liking,” and the ayahuasca was well tolerated. However, one subject who took the medium dose experienced an intensely dysphoric reaction with transient disorientation and anxiety that lasted 20 minutes without the need for medication [40, 47]. In another clinical study using fMRI and involving 10 subjects, effects were significant only at 40- and 80-minutes post‐intake in the brief psychiatric ratings scale (BPRS) used to detect psychotic symptoms and the Young Mania Rating Scale (YMRS) used to measure mania symptoms, but subjects were not incapacitated from performing the imagery task involved [36]. A recent controlled clinical trial studying the effects of ayahuasca on the recognition of facial expressions of emotions in naive healthy volunteers did not find differences in clinical variables between ayahuasca and placebo [43].

One problem in characterizing the effects of ayahuasca is the variable composition of the brew. Although the most common combination is *B*. *caapi* and *P*. *viridis*, in Colombia and parts of Ecuador instead of *P*. *viridis*, *D*. *cabrerana* is used. There are no studies comparing both types of ayahuasca. Furthermore, ayahuasca is not always cooked in the same way and plant constituents can vary based on factors such as the age of the plant, soil of cultivation, and time of collection [59]. Ayahuasca also contains various other plant constituents such as flavonoids and terpenes. A recent study quantified about 2,000 components in a final ayahuasca brew utilising the traditional plants [60]. To make things even more complicated, now in Europe and Australia it is becoming popular to substitute other non-traditional plants containing DMT (like Mimosa tenuiflora or Acacia) with others that contain harmines (like Peganum harmala) [11, 16], which vary in the active constituent levels as well as containing other non-alkaloids compounds that differ from the traditional plants. Observational research should acknowledge and ideally account for such differences.

In sum, although the types of general side effects of ayahuasca are relatively well known, their rate of occurrence, severity, and persistence seems to differ depending on the degree of control in the study, the knowledge of medical and psychological antecedents, the setting where ayahuasca was used, composition of the decoction, and demographic characteristics. Due to the relatively small samples sizes of the different studies that have focused on ayahuasca’s adverse side effects, it is difficult to establish relationships between reported side effects and clinical, personal, social, and contextual variables. The present study responds to these gaps using a very large sample of ayahuasca users. We analyse the frequency of ayahuasca’s adverse effects, as well as relationships between reported adverse effects and history of ayahuasca use, the reported strength of the acute spiritual experience, clinical, sociodemographic, and contextual variables.

## Methods

### Sample

Participants were drawn from 10,836 individuals who participated in the Global Ayahuasca Project, an online survey performed between 2017 and 2019. The Global Ayahuasca Project, formally known as the “Global survey of ayahuasca drinking: practices, beliefs and reported effects on health and wellbeing” is a multidisciplinary research project based at the University of Melbourne that has been undertaken in partnership with an international team of researchers from Australia, Brazil, Spain, the Czech Republic, and Switzerland and has collected detailed data regarding the consumption of ayahuasca in different contexts around the world, including motivations and contexts of drinking, reported effects on health and well-being and adverse effects (a copy of survey can be obtained from the corresponding author). The survey was released in six languages (English, Portuguese, Spanish, German, Italian, and Czech) and collected data from participants from more than 50 countries who were aged at least 18 years of age and had used ayahuasca on one or more occasions. The study was approved by the University of Melbourne Human Research Ethics Committee (HREC number 1545143.3) and all participants were adults who provided their written informed consent via an initial question in the online survey. Participants could not proceed if consent was not provided.

Survey participation was promoted via websites and email invitations from relevant organizations, ayahuasca retreat centres, and ayahuasca churches, online groups and forums, via Facebook, and flyers at conferences and events. The recruitment text said: “The project aims to increase understanding of the drinking of ayahuasca in different contexts around the globe and will explore motivations and contexts of drinking, reported effects on health and well-being, and any potential risks” (https://www.globalayahuascaproject.org/). No financial incentives were offered. Data was cross-checked to remove suspected duplicate responses, while data from partially completed surveys was retained. Due to the obscure nature of the ayahuasca-using population in many countries (where this practice is either prohibited or where its legal status remains unclear), a non-random sampling method was chosen. Context was defined by asking whether they had consumed ayahuasca in a ceremony with “one or more traditional shaman” (traditional); with the UDV, Santo Daime or Barquinha churches (ayahuasca church); or with non-traditional, mixed traditional/non-traditional, and no guide or no ceremony/ritual, which was included as “non-supervised context”. In this study, only participants without missing responses to the Ayahuasca Adverse Effects section of the survey were included. This resulted in a study sample of 8,216 for the adverse physical effects and 7,839 participants for the adverse mental health effects studied. No significant differences were observed between excluded participants and those included in the adverse physical effect sample in terms of ayahuasca doses/year, lifetime ayahuasca use, last year ayahuasca use, or sex distribution (all *p*s ≥ .10); however, the included participants were older [40.7 (12.2) vs. 38.9 (12.2); p < .001]. Notably, in the adverse mental health effects sample, the included participants used a significantly higher number of doses/year [1.7 (.02) vs. 1.6 (.03); *p* = .03], reported greater lifetime ayahuasca use [3.5 (2.2) vs. 3.3 (2.3); *p* = .002], and were older than the excluded participants [40.7 (12.2) vs. 39.1 (12.3); *p* < .001].

### Questionnaire

Demographic information such as age, sex, highest level of education, and country of residence was obtained from participants, in addition to their lifetime history of mental health diagnoses and detailed ayahuasca drinking history, including frequency, patterns, and contexts of use. The intensity of the acute subjective spiritual experience was evaluated via a modified version of the nine-item Short Index of Mystical Orientation (SIMO) [61] (see [5] for more information).

Acute adverse physical health effects were obtained via a checkbox question with 10 specified options, plus “other”, asking if any of these physical health issues had ever been experienced “during or soon after an ayahuasca ceremony/session”. Individuals selecting any physical health issue were then asked if medical attention was required for that issue. A subsequent question asked about short to medium-term mental health, emotional or perceptual changes (in the weeks or months after consumption) and was based on the PHQ-4 (Patient Health Questionnaire for Depression and Anxiety, four items; [62], plus six additional items derived from the ayahuasca literature. This question asked respondents “In the weeks or months after your ayahuasca ceremonies/sessions have you ever experienced an increase in any of the following”, with responses using a modified version of the PHQ-4 four-point scale (Not at all, Slightly, Moderately, Very Much). Note this question did not specifically describe these items as adverse effects. Individuals reporting an increase on any item were asked if they required “support from a psychiatrist, physician, therapist, counsellor, or alternative health practitioner to cope with these feelings/experiences?” and then if they “believe the feelings were/are part of a positive process of growth or integration?” (No/Somewhat/Yes/Not sure).

### Statistical analysis

Before the study analysis, a preliminary analysis was performed to reduce the number of analysis of the study. As both, the physical and mental adverse effects identified are heterogeneous a principal component analyses were performed with each adverse effect types to study the adverse effect factor structure. Based on between adverse effects correlations, the factorial analysis performed regarding the ayahuasca adverse physical effects was performed using a varimax rotation procedure, and the mental health adverse effects was performed using a promax rotation procedure.

To test for the frequency and prevalence of each specific adverse effect, the presence of adverse physical and mental health effects was included in each category, and the factors related to each type of adverse effect is presented. These results are presented for the full sample and for participants who had only drunk ayahuasca once. Moreover, as the adverse mental health effects were measured using a 4-point scale, the frequency at which participants reported severe adverse mental health effects is also reported. Severe adverse mental health effects were assumed when the participants responded that the item had increased “very much”. Finally, the frequency of participants needing medical attention or professional support for their physical health and mental health adverse effects respectively, as well as the frequency of participants with less than a week duration of their mental health adverse effects is reported.

To test for adverse effects associated with a history of ayahuasca use, clinical, spiritual experience, and context of consumption variables, logistic regressions analyses were performed. The same analysis procedure was used to test for the presence of adverse physical and mental health effects, and for each of the factors observed in the preliminary analyses. The history of ayahuasca use variable’s (age of initial use, average dose per year, lifetime use, and last year use), the clinical variables’ (history of anxiety disorder, depressive disorder, substance use disorders, alcohol use disorder, and number of physical health conditions), acute spiritual experience (SIMO score), and context of consumption (religious, traditional shamanic, non-traditional supervised, non-supervised contexts) association with the adverse effects was analysed by odds ratio (OR), 95% confidence interval (CI), and the significance of the variables in the model, and the β was also included to analyse the association direction. The regression analysis performed for each dependent variable (Psychical and Mental Health adverse effects and the factors of each adverse effects) was controlled for sociodemographic variables. Only significant controlled variables are included as a footnote in each table. The comparison category for the independent categorial variables were female for sex; the presence of each disorder studied; and religious context for context. Finally, as three of the independent variables (ayahuasca doses/year, lifetimes ayahuasca use, and last year ayahuasca use) showed great positive asymmetry, these variables were normalized using Ln(x) transformation. Statistical significance was regarded as ≤ .05.

## Results

### Sample characteristics

As responders with missing values in the adverse effects section were excluded from the analysis, the sample size to study Physical and Mental Health adverse effects were different. Eight thousand two hundred and sixteen participants were included in the analysis of the adverse physical effects and 7,839 in the analysis of the adverse mental effects. More than 46.0% were female, with an average age over forty. The majority of the participants had a University degree, and were most commonly married and from Brazil. Finally, the participants had more than 30 lifetime ayahuasca uses and ayahuasca was used principally in a religious context (Table 1).

**Table 1:** Samples characteristics ^a^^,^ ^b^.

	Physical adverse effects (8,216)^d^		Mental Health adverse effects (7,839)^e^	
	N	%	n	%
**Sociodemographics**				
Female	3,821	46.5	3,674	46.9
Mean age (SD)	40.74	(12.18)	40.74	(12.16)
Universitary Education	5,223	63.6	5,007	63.9
Active professionals	3,617	44.0	3,472	44.3
Married	3,068	37.3	2,981	38.0
**Region of residence**				
Brazil	3,832	46.6	3,722	47.5
Other Latin America	410	5.0	388	4.9
Europe	1,998	24.3	1,894	24.2
North America	1,244	15.1	1,179	15.0
Austraia & NZ	340	4.1	318	4.1
Asia and Middle East	57	.7	51	.7
**Clinical**				
Anxiety disorder	1,092	13.3	1,050	13.4
Depressive Disorder	1,517	18.5	1,469	18.7
Substance use disorder	770	9.4	745	9.5
Alcohol use disorder	769	9,4	750	9.6
Any physical condition	1,752	21.3	1,691	21.5
**Ayahuasca use history**				
Mean (SD) age of Ayahuasca use onset	31.06	(12.63)	31.01	(12.65)
Lifetime ayahuasca use^c^				
2 uses or less	1,175	13.7	1,067	13.6
3–8 uses	1,629	19.8	1,554	19.8
9–30 uses	1,349	16.4	1,291	16.5
More than 30	3,697	45.0	3,584	45.7
Last year ayahuasca use^c^				
5 uses or less	3,487	42.4	3,307	55.6
6–30 uses	2,516	30.6	2,443	31.2
31–250 uses	1,672	20.4	1,633	20.8
More than 250 uses	36	.4	36	.5
**Context of ayahuasca use**				
Religious	3,774	45.9	3,661	46.7
Traditional shaman	1,656	20.2	1,579	20.1
Non-traditional	2,090	25.4	1,997	26.1
Non-supervised context	442	5.4	410	5.4

^**a**^ Percentages may not sum to 100.0% due to rounding

^b^;”(n)” indicates valid (non-missing) sample for each item

^c^: variable category represented the variable percentile

^d^ participants with non-missing response to the Adverse physical effects

^e^ participants with non-missing response to the adverse mental effects.

### Preliminary analyses

Results of the preliminary analyses are presented in Tables A-D in S1 File, with the more relevant results reported here to clarify the subsequent results. As expected, correlations between adverse physical effects were low, ranging from .04 to .24. Both the Kaiser-Meyer-Olkin (.75) and the sphericity Bartlett tests (χ^2^_(45)_ = 4,444.4; *p* < .001) showed appropriate values for a principal component analysis. The results showed 3 factors (eigenvalues ≥ 1.05) with an explained variance of 21.4%, 10.5%, and 10.5%, respectively (Table A in S1 File). The first factor included abdominal pain, vomiting/nauseas, breathing difficulties, chest pains, and headache (general symptom factor); the second one, stiff/swollen joints, aching muscles, and coughing/wheezing (arthromyalgical factor); and the last one, fits or seizures and fainting (neurological factor) (Table B in S1 File).

Results for the factorial analysis of adverse mental health effects are presented in Tables C and D in S1 File. The results of Kaiser-Meyer-Olkin (.87) and Bartlett sphericity (χ^2^_(45)_ = 30,112.6; *p* < .001) tests also showed that principal component analysis was appropriate. The results showed two factors with eigenvalues greater than 1.0, and the explained variance for each factor was 44.5% and 14.3%, respectively. The first factor included feeling nervous, anxious, uncontrolled worrying, little interest or pleasure in doing things, feeling disconnected or alone, difficulties knowing what is real and not real, and having nightmares or disturbed thoughts (emotional-cognitive factor), while the second factor included hearing or seeing things that others do not hear or see, visual distortions, and feeling attacked by the spirit world (psychotomimetic factor).

### Ayahuasca’s adverse effects

Adverse effects were frequently reported. Moreover, in the subsample of those participants who had drunk ayahuasca only once, although the frequency of each adverse effect was lower, the pattern of the adverse effects’ frequency observed was quite similar to that observed for the full sample (Table 2). Adverse physical effects occurring on at least one occasion was reported by 5,742 participants (69.9%) and adverse mental health effects on at least one occasion by 4,341 participants (55.4%). Among the adverse physical effects, general symptom effects were more frequently observed (5,603; 68.2%), while arthromyalgical and neurological effects were less frequently observed (arthromyalgical: 883; 10.7%; neurological: 416; 5.1%). Regarding the specific adverse physical effects studied, vomiting/nausea was very frequent in the sample (5,097; 62.0%), while the frequency of the other effects ranged between 1.3% and 17.8% (fits or seizures: 106; headache: 1,460). Also with a low frequency, fainting was reported by 4.1% of the sample (335). However, although 69.9% of the sample reported some adverse physical effects, 30.1% (n = 2,291) did not report any adverse effect, and 39.9% (n = 3,126) only reported one adverse effect. Finally, only 132 (2.3%, n = 5,775 of participants who answered the question) of the participants reported physical health adverse effects reportedly requiring medical attention.

**Table 2:** Frequency of participants’ reported adverse physical effects (n = 8,216).

	Total sample (8,216)	Single ayahuasca use participants (598)
	n %	n (%)
**Adverse physical effect** ^1^ ^,^ ^2^	5,742 (69.9)	334 (55.9)
**General symptom adverse effect** ^1^ ^,^ ^3^	5,603 (68.2)	321 (53.7)
Vomiting/nausea	5,097 (62.0)	281 (47.0)
Headache	1,460 (17.8)	57 (9.5)
Abdominal pain	1,052 (12.8)	31 (5.2)
Breathing difficulties	599 (7.3)	33 (5.5)
Chest pains	384 (4.7)	14 (2.3)
**Arthromyalgical adverse effect** ^1^ ^,^ ^4^	883 (10.7)	39 (6.5)
Aching muscles	617 (7.5)	29 (4.8)
Coughing/wheezing	273 (3.3)	5 (0.8)
Stiff/swollen joints	182 (2.2)	12 (2.0)
**Neurological adverse effect** ^1^ ^,^ ^5^	416 (5.1)	20 (3.3)
Fainting	335 (4.1)	13 (2.2)
Fits or seizures	106 (1.3)	7 (1.2)

^1^ Reported presence of some of the adverse physical effects studied

^2^ adverse physical effects range (0–10) and median (1.0)

^3^ adverse psychophisical effects range (0–5) and median (1.0)

^4^ adverse arthromyalgic effects range (0–3) and median (0.0)

^5^ adverse neurological effects range (0–2) and median (0.0).

The results for adverse mental health effects are presented in Table 3. Adverse mental health effects on at least one occasion were also highly reported (4,341; 55.4%). Specifically, emotional-cognitive effects (3,293; 42.0%) and altered perceptions effects (3,004; 38.3%) were most highly reported. The adverse mental health effects most frequently reported by participants were “hearing or seeing things that others do not hear or see” (2,236; 28.5%) and “feeling disconnected or alone” (1,650; 21.0%). Meanwhile, the least observed adverse mental health effects were “difficulty knowing what is real and not real” (1,011; 12.9%) and “little interest or pleasure in doing things” (1,160.0; 14.8%). However, although the presence of adverse mental health effects in the sample was high, 44.6% did not report any adverse mental health effects, and the frequency of specific *severe* adverse mental health effects was only higher than 4.0% for visual distortions (4.4%; 342). Moreover, for the majority of participants reporting seven of the 10 adverse mental health effects, the duration was less than one week. For the other three adverse effects 44%-49% reported a duration of less than a week. There were 11.9% (n = 4,315) that reported needing professional support for the adverse effects that they had experienced. Finally, of all respondents identifying adverse mental health effects 87.6% believed that these were completely (76.3%) or somewhat (11.3%) “part of a positive process of growth or integration”, while 3.9% felt they were not and 8.6% were unsure.

**Table 3:** Frequency of participants’ reported adverse mental health effects.

	Total sample (7,839)			Single ayahuasca use participants (565)
	Adverse effects	Adverse effects duration	Severe adverse effects	Adverse effects
	n (%)	n (%)*	n (%)	n (%)
**Adverse mental health effects** ^1^ ^,^ ^2^	4,341 (55.4)			289 (51.2)
**Emotional-cognitive adverse effects** ^1^ ^,^ ^3^	3,293 (42.0)			243 (43.0)
Feeling disconnected or alone	1,650 (21.0)	788 (49.0)	233 (3.0)	120 (21.2)
Nightmares, disturbing thoughts, feelings, or sensations	1,506 (19.2)	611 (52.7)	175 (2.2)	88 (15.6)
Feeling nervous, anxious, or on edge	1,483 (18.9)	833 (57.2)	247 (3.2)	110 (19.5)
Feeling down, depressed, or hopeless	1,300 (16.6)	643 (50.6)	149 (1.9)	79 (14.0)
Not being able to stop or control worrying	1,201 (15.3)	665 (56.7)	185 (2.4)	85 (15.0)
Little interest or pleasure in doing things	1,160 (14.8)	595 (52.3)	134 (1.7)	76 (13.5)
Difficulty knowing what is real and not real	1,011 (12.9)	509 (51.8)	167 (2.1)	84 (14.9)
**Altered perception adverse effects** ^1^ ^,^ ^4^	3,004 (38.3)			159 (28.1)
Hearing or seeing things that other people do not hear or see	2,236 (28.5)	646 (44.1)	251 (3.2)	76 (13.5)
Feeling “energetically attacked” or a harmful connection with a “spirit world”	1,186 (14.9)	579 (48.9)	191 (2.4)	49 (8.7)
Visual distortions	2,236 (15.1)	1330 (60.7)	342 (4.4)	117(20.7)

* Calculated for those who had been reported Adverse Effects. Missing data not included

^1^ reported presence of some of the adverse mental health effects studied

^2^ adverse mental health effects total score mean (12.78), S.D. (4.3) and range (0–30)

^3^ adverse emotional effects total score mean (1.76), S.D. (3.3), range (0–21)

^4^ adverse altered perception effects mean (1.02), S.D. (1.7), range (0–9).

### Associations between adverse physical effects, history of use, and clinical variables

The analysis of ayahuasca’s adverse physical effects’ relationship with history of ayahuasca use, clinical, acute spiritual experience, and context of ayahuasca use variables are shown in Table 4. Between the independent variables studied, adverse physical effects have a higher significant and positive relationship with lifetime ayahuasca use, substance use disorder, physical health conditions and with non-supervised context (*ps* ≤ .05) compared to a religious context. Moreover, although to a lesser extent, age of first ayahuasca use and last year ayahuasca uses (*ps* ≤ .05), also increased the risk of adverse physical effects. Only doses/year (*p* = .05) was negatively related to adverse physical effects. In relation to the controlled sociodemographic variables (see footnotes Table 4), being female (*p* = .05) and having a higher education degree increased the risk of adverse physical effects (*ps* ≤ .05), while a significant negative relationship was observed with age at survey date (*p* < .001).

**Table 4:** History of ayahuasca use and medical status variables’ relationships with the presence of adverse physical effects.

	F062	*p*	OR	OR (95% C.I.)	
Age of initial use	.030	< .001	1.03	1.02	1.04
Doses/year	-.138	.04	.87	.76	.99
Lifetime use	.226	< .001	1.25	1.18	1.41
Last year use	.090	.05	1.09	1.00	1.20
Anxiety disorder	.187	.06	1.20	.99	1.47
Depressive disorder	.023	.80	1.02	.86	1.22
Substance use disorder	.239	.05	1.27	1.00	1.61
Alcohol use disorder	-.026	.82	.97	.78	1.22
Physical health conditions	.167	.002	1.18	1.06	1.32
Acute spiritual experience	.003	.17	1.00	1.00	1.01
Context					
Religious context	–	–	1		
Traditional shaman context	-.170	.11	.84	.68	1.04
Non-traditional context	-.041	.68	.96	.79	1.17
Non-supervised context	.374	.03	1.45	1.04	2.02

^1^ Significant controlled variables: female: β = .242; *p* < .001; OR=1.27 (1.12-1.44); age at survey day: β= -.033; *p* < .001; OR=.97 (.95-.98); education: Diploma/advance diploma β = .45; *p* =.05; OR=1.57 (1.01-2.47); undergraduate/Bachelor β = .63*; p*=.005; OR=1.88 (1.21-2.92); Master’s degree: β = .71; *p* = .002; OR=2.03 (1.30-3.15); PhD degree: β = 1.05; *p* < .001; OR=2.86 (1.69-4.85).

The results for the relationships of adverse physical effects factors (see preliminary results; general symptom, arthromyalgical, and neurological) with the studied variables show a more detailed picture (Table 5). While the results observed for the general symptom factor seem similar to those observed in the general analysis, those observed for the arthromyalgical and neurological factors show remarkable specificities. Regarding the adverse general symptoms, few differences from the general model were observed. That is, a higher risk of general adverse effects was positively related to older age of first ayahuasca use (*p* < .001), a greater number of lifetime uses (*p* < .001) and last year uses (*p* = .01), the presence of a physical health condition (*p* = .003), and especially with a non-supervised context of ayahuasca use (*p* = .01) compared with religious context. However, compared to the significant relationship observed in the general model, substance use disorder (*p* = .07) only approached significance. Moreover, as it was observed in the general model, fewer doses/year increase the risk of adverse general symptoms (*p* = .03). Moreover, the same pattern of significant control variables observed in the general analysis was associated with the general symptom factor (see footnotes Table 5).

**Table 5:** History of ayahuasca use and medical status variables’ relationship with the presence of adverse physical effects for each factor.

	β	*p*	OR	OR (95% C.I.)	
**Adverse general symptom effects**					
Age of initial use	.028	< .001	1.03	1.01	1.04
Doses/year	-.145	.03	.86	.76	.98
Lifetime use	.235	< .001	1.26	1.13	1.42
Last year use	.108	.01	1.11	1.02	1.21
Anxiety disorder	.181	.07	1.20	.99	1.45
Depressive disorder	.042	.63	1.04	.88	1.24
Substance use disorder	.210	.07	1.23	.98	1.55
Alcohol use disorder	.044	.70	1.04	.84	1.31
Physical health conditions	.161	.003	1.17	1.06	1.30
Acute spiritual experience	.002	.356	1.00	1.00	1.01
Context					
Religious context	–	–	1		
Traditional Shaman context	-.099	.34	.91	.74	1.11
Non-traditional context	.056	.57	1.06	.87	1.28
Non-supervised context	.399	.01	1.49	1.08	2.05
**Adverse arthromyalgical effects**					
Age of initial use	.029	.01	1.03	1.01	1.05
Doses/year	-.122	.24	.88	.72	1.08
Lifetime use	.240	.008	1.27	1.06	1.52
Last year use	.053	.39	1.05	.93	1.19
Anxiety disorder	.236	.05	1.27	.99	1.61
Depressive disorder	.149	.19	1.16	.93	1.45
Substance use disorder	.255	.08	1.29	.97	1.72
Alcohol use disorder	-.322	.04	.72	.53	.99
Physical health conditions	.383	< .001	1.47	1.30	1.65
Acute spiritual experience	.016	< .001	1.02	1.01	1.02
Context					
Religious context	–	–	1		
Traditional Shaman context	.694	< .001	2.00	1.50	2.66
Non-traditional context	.649	< .001	1.91	1.46	2.50
Non-supervised context	.707	< .001	2.03	1.38	2.98
**Adverse neurological effects**					
Age of initial use	.026	.09	1.03	1.00	1.06
Doses/year	-.272	.07	.76	.57	1.02
Lifetime use	.330	.01	1.39	1.08	1.80
Last year use	-.095	.25	.91	.77	1.07
Anxiety disorder	-.244	.21	.78	.53	1.15
Depressive disorder	-.202	.23	.82	.59	1.14
Substance use disorder	.256	.22	1.29	.86	1.94
Alcohol use disorder	.038	.86	1.04	.69	1.57
Physical health conditions	.312	< .001	1.37	1.16	1.61
Acute spiritual experience	.018	< .001	1.02	1.01	1.03
Context					
Religious context	–	–	1		
Traditional Shaman context	.055	.79	1.06	.71	1.57
Non-traditional context	.035	.85	1.03	.71	1.50
Non-supervised context	.164	.56	1.18	.68	2.04

^1^ Significant controlled variables: Adverse psychophisical effects: female: β = .22; *p* < .001; OR = 1.24 (1.10–1.40); age at survey day: β = -.03; *p* < .001; OR = .97 (.95-.98); education: undergraduate/Bachelor β = .55; *p* = .01; OR = 1.74 (1.12–2.70); Master’s degree: β = .62; *p* = .006; OR = 1.87 (1.20–2.90); PhD degree: β = .79; *p* = .003; OR = 2.20 (1.31–3.68). Adverse arthromyalgical effects: female: β = .20; *p* = .02; OR = 1.23 (1.03–1.46); age at survey day: β = -.03; *p* = .004; OR = .97 (.95-.99); married β = -.40; *p* = .001; OR = .67 (.53-.84); divorced or separated: β = -.77; *p* < .001; OR = .46 (.31-.68). Adverse neurological effects: female: β = .31; *p* = .01; OR = 1.36 (1.07–1.73); partner: living with partner β = -.41; *p* = .03; OR = .66 (.45-.97); married β = -.59; *p* < .001; OR = .55 (.40-.76).

The results for adverse arthromyalgical effects are presented in Table 5. Regarding the adverse arthromyalgical effects, a physical health condition, anxiety disorder, higher lifetime use and an older age of initial ayahuasca use (*ps* ≤ .01) increased the risk of adverse effects. However, having an alcohol use disorder (*p* = .04) was significantly and negatively related to adverse arthromyalgical effects. On the other hand, while acute spiritual experience slightly increases the risk of adverse effects (*p* < .001), compared with use in a religious context, all the other contexts of use studied significantly increased the risk of adverse arthromyalgical effects (*ps* < .001). Finally, among the control variables, a higher risk of adverse effects was positively associated with being female (*p* = .02) and negatively associated with participants’ age, being married, and being divorced or separated (*p* ≤ .001) at the survey date (see footnotes Table 5).

Finally, experiencing any adverse neurological effects was positively associated with having a physical health condition and number of lifetime ayahuasca uses (*p* ≤ .01). However, a slight association was also observed with acute spiritual experience (*p* < .001). No significant associations were observed between adverse neurological effects and context of use (all *ps* ≥ .56). Related to the control variables, being female (*p* = .01) significantly increase the risk of adverse neurological effects, while living with partner and being married (*p* ≤ .03) were negatively associated (Table 5).

### Association with ayahuasca’s adverse mental health effects

The results for the relationships between adverse mental health effects and the ayahuasca history of use, clinical, spiritual experience and context of use variables are presented in Table 6. Having an anxiety disorder, having a physical health condition, and the strength of the acute spiritual experience (*ps ≤* .04) all significantly increased the risk of adverse mental health effects, as did use in all non-religious contexts: traditional shaman, non-traditional, and non-supervised context (*p* ≤ .02) (Table 6).

**Table 6:** History of ayahuasca use and medical status variables’ relationship with adverse mental health effects for each factor.

	β	*p*	OR	OR (95% C.I.)	
**Adverse mental health effects**					
Age of initial use	.012	.08	1.01	1.00	1.02
Doses/year	.009	.89	1.01	.89	1.14
Lifetime use	.027	.64	1.03	.92	1.15
Last year use	-.008	.85	.99	.91	.108
Anxiety disorder	.234	.01	1.26	1.05	1.51
Depressive disorder	.110	.18	1.12	.95	1.31
Substance use disorder	.016	.88	1.02	.82	1.25
Alcohol use disorder	.090	.39	1.09	.89	1.34
Physical health conditions	.101	.04	1.10	1.01	1.22
Acute spiritual experience	.016	< .001	1.02	1.01	1.02
Context					
Religious context	–	–	1		
Traditional Shaman context	.230	.02	1.26	1.03	1.53
Non-traditional context	.453	< .001	1.57	1.31	1.89
Non-supervised context	.538	< .001	1.71	1.28	2.29
**Adverse emotional-congnitive effects**					
Age of initial use	.012	.09	1.01	1.00	1.03
Doses/year	.014	.83	1.01	.89	1.15
Lifetime use	.008	.89	1.01	.90	1.13
Last year use	-.052	.22	.95	.87	1.03
Anxiety disorder	.357	< .001	1.43	1.20	1.70
Depressive disorder	.146	.07	1.16	.99	1.35
Substance use disorder	-.010	.93	.99	.80	1.22
Alcohol use disorder	-.014	.89	.99	.80	1.21
Physical health conditions	.062	.20	1.06	.97	1.17
Acute spiritual experience	.011	< .001	1.01	1.01	1.01
Context					
Religious context	–	–	1		
Traditional Shaman context	.193	.05	1.21	1.00	1.47
Non-traditional context	.454	< .001	1.57	1.31	1.89
Non-supervised context	.447	.002	1.56	1.18	2.06
**Adverse psychotomimetic effects**					
Age of initial use	.021	.002	1.02	1.01	1.03
Doses/year	-.088	.19	.92	.80	1.04
Lifetime use	.173	.003	1.19	1.06	1.33
Last year use	.025	.54	1.03	.94	1.11
Anxiety disorder	.134	.13	1.14	.96	1.36
Depressive disorder	.141	.08	1.15	.98	1.35
Substance use disorder	.161	.12	1.17	.96	1.44
Alcohol use disorder	.046	.65	1.05	.85	1.28
Physical health conditions	.174	< .001	1.19	1.08	1.31
Acute spiritual experience	.023	< .001	1.02	1.02	1.03
Context					
Religious context	–	–	1		
Traditional Shaman context	.248	.01	1.28	1.05	1.56
Non-traditional context	.307	.001	1.36	1.13	1.63
Non-supervised context	.419	.003	1.52	1.15	2.01

^**1**^ Significant controlled variables: Adverse mental health effects: female: β = .16; p = .006; OR = 1.17 (1.05–1.32); age at survey day: β = -.02; p = .007; OR = .98 (.97-.99); married: β = - .38; p < .001; OR = .68 (.58-.79); Adverse emotional-cognitive effects: female β = .16; p = .006; OR = 1.18 (1.05–1.32); age at survey day: β = -.02; p = .002; OR = .98 (.96-.99); married β = - .44; p < .001; OR = .64 (.55-.74); Adverse psychotomimetic effects: female β = .18; p = .002; OR = 1.19 (1.07–1.34); age at survey day: β = -.02; p = .001; OR = .98 (.96-.99); married β = - .27; p = .001; OR = .76 (.65-.89).

Regarding each of the factors of the adverse mental health effects, a different pattern of relationships emerged. Adverse emotional-cognitive effects were significantly associated with having an anxiety disorder (*p* < .001), the acute spiritual experience (*p* < .001), and consumption outside a religious context (p ≤ .05). However, higher risk of adverse psychotomimetic effects was associated with an older age of initial ayahuasca use, a higher number of lifetime uses, having a physical health condition, the acute spiritual experience, and a non-religious context of use (*p* ≤ .05). Finally, in relation to the sociodemographic control variables, the same pattern of relationships was observed for the adverse mental health, adverse emotional-cognitive, and psychotomimetic effects. While being female significantly increase the risk of adverse mental health effects (*ps* = .003), a younger age of initial use (*ps* = .007), and being married *ps* ≤ .001) significantly reduce the risk of adverse mental health effects (Table 6).

### Supplementary context analysis

As compared with religious context of ayahuasca use, other contexts of use significantly increase the risk of ayahuasca adverse effects, especially arthromyalgical and mental health adverse effects, supplementary analysis were performed to test between context of use differences in the independent variables studied (see Table E in S1 File). Between contexts of use significant differences were observed in all the independent variables studied (*p* ≤ .004) with the only exception of alcohol use disorder (*p* = .08).

Respondents who consumed ayahuasca in religious contexts reported a higher number of dose/year (To test between group differences, an ANOVA with the ln transformed variables was used (see Statistical Analysis section)) (*p* < .001), greater lifetime use^3^ (*p* < .001), than the others context studied. Contrary, lower frequency of anxiety disorder (*p* < .001), depressive disorder (*p* < .001), and number of physical health conditions (*p* < .001) were observed in religious context compared with the other context studied. Moreover, participants’ age of ayahuasca use onset in religious context was lower than in the other context of use (*p* < .001) and it was observed lower frequency of substances use disorder in religious context compared with non-supervised context (*p* < .001).

Without including the religious contexts, non-supervised contexts showed a higher number of ayahuasca dose/year^3^ (*p* ≤ .02), higher lifetime use (*p* < .001), and higher last year use (*p* < .001) than that observed in traditional and non-supervised contexts of use. A higher frequency of substance use disorders was observed in non-supervised contexts compared with all other contexts of use, including religious use (*p* < .001).

Finally, while the 84.0% of the religious context ayahuasca users were Brazilian, it was observed that the majority of the ayahuasca users in the others context of use (higher than 37.0%) were European (*p* < .002) (see Table E in S1 File).

## Discussion

The ritual use of ayahuasca is expanding internationally; its neuropharmacology is well characterized, e.g. [44, 45, 63], its long-term safety has been well-studied, e.g. [31, 64, 65] and both prospective longitudinal studies, e.g. [21, 23] and controlled trials, e.g. [41, 45] show promising therapeutic outcomes. However, evidence on relationships between adverse effects and individuals’ history of ayahuasca use, and clinical, sociodemographic, contextual, and ayahuasca spiritual experience variables have not been previously explored. As expected, in this study using a large online survey sample, it was found that adverse ayahuasca effects are frequent, but generally mild and transient. The most prevalent adverse physical effect was vomiting/nausea, and the most prevalent adverse mental health effect was reported in the domain of altered perception. A small number of participants who experienced adverse effects needed medical attention or professional mental health support. While adverse physical effects were principally associated with participants’ physical health antecedents and higher last year use, adverse mental health effects were related to participants’ previous anxiety disorder, higher doses/year, and lower lifetime use. Furthermore, both adverse physical and mental effects were significantly associated with non-supervised and non-traditional supervised contexts, while consumption in a religious context was associated with fewer adverse effects than other contexts.

### Adverse physical effects

As noted above, the most frequently reported adverse physical health effect was vomiting/nausea (68.2%), while the frequency of other adverse effects was 17.8% (headache) or lower. It is important to clarify that vomiting/nausea is considered a normal effect of ayahuasca for experienced users. In the case of traditional ayahuasca ceremonies and even in non-traditional ceremonies, not only is vomiting/nausea not considered an adverse effect, but it is even sought out for its purging and perceived spiritual cleansing benefits [46]. We did not collect data regarding how long the reported physical adverse events lasted nor the degree of severity. For the subsample of those participants who had drunk ayahuasca only once, although the frequency of each adverse effect was lower, the pattern of the adverse effects’ frequency observed was quite similar to the observed in all the sample. However, 2.3% of participants reportedly required medical attention for the physical adverse effects experienced. These results are consistent with previous studies, with regular users reporting that most adverse physical effects seem to not be serious and do not compromise health [52, 57].

Although the majority of adverse physical effects did not seem to be serious, the frequency of adverse effects related to the neurological factors (fainting, 4.1%, and fits or seizures, 1.3%) were reported for about 5.0% of the participants. However, given the self-report nature of our data we cannot confirm whether these were actually of neurological or ‘psychological’ (pseudo-neurological) origin. Although neurological adverse effects are poorly reported in the literature and were rarely reported in this study, it is important to take them into account because of their potential severity. Consistent with previous results, Gómez-Sousa et al. (2021) [50] reported 2 cases (from a sample of 40) of loss of consciousness, one of them with seizures (a woman who had a previous history of epilepsy). Classical hallucinogens, including DMT [66], bind with 5-HT_2A_ receptors, inducing the release of glutamate, which, theoretically, may eventually lead to convulsions and seizures in neurologically vulnerable individuals. In fact, this is an effect that occasionally occurs in ayahuasca ceremonies, according to field work informants. In traditional settings, this is explained as a special kind of spiritual phenomena, but it is important to be alert to the risk of seizures in individuals with a history of epilepsy or any kind of brain disease. Interestingly, as will be commented on later, although previous physical health conditions increased the likelihood of some adverse physical effects (OR = 1.18), their presence increases the risk of adverse neurological effects to a greater extent (OR = 1.37).

Examination of the relationships between ayahuasca’s adverse physical effects and history of ayahuasca use, clinical variables and sociodemographic variables identified that adverse effects were more likely to be observed in participants with higher previous year use, greater lifetime use, older age at initial ayahuasca use, and in those with a higher number of previous physical health conditions, a comorbid anxiety disorder, or previous diagnosis of a substance use disorder. Moreover, while being female, younger, and having a higher academic degree increased the likelihood of reporting adverse physical effects, being married at the survey date was associated with fewer adverse physical effects. Finally, adverse physical effects were more frequently associated with a non-supervised context of ayahuasca use.

When the adverse physical effects were analysed by factors (general symptom, arthromyalgical, and neurological; see preliminary results), a different pattern of relationships emerged. While the variables associated with adverse general symptom effects were similar to those observed in the general analysis, some important changes were observed regarding the adverse arthromyalgical and neurological effects. The adjusted model indicated that adverse arthromyalgical effects were more likely to be observed in those with a higher number of previous physical health conditions, higher lifetime ayahuasca use, and older age at initial ayahuasca use, while being less likely in those with a previous diagnosis of alcohol use disorder. The adverse neurological effects, in the adjusted model, were only more likely to be observed in participants with higher lifetime ayahuasca use and a higher number of previous physical health conditions. However, higher last year use decreased the likelihood of adverse neurological effects. Finally, although adverse arthromyalgical effects were more likely to be observed in younger participants, who experienced a more intense acute spiritual experience, and those who used ayahuasca in a non-traditional supervised context, adverse neurological effects were associated with being female and the acute spiritual experience, but not with context of use.

Three considerations should be mentioned based on the above. The special case of the neurological factor being related to higher lifetime use seems to suggest that individuals continue using ayahuasca despite having experienced some episode and, thus, it is not necessary to have a history of seizures to suffer a seizure. This is also in accordance with our observations in the field along with our previous research: sometimes neurological effects are transitory and do not prevent people from continued use of ayahuasca because they are perceived as resulting from a kind of ‘spiritual force’. In relation to context of use, there was no association with adverse neurological effects, all non-religious contexts (traditional, non-traditional, and non-supervised) were positively associated with arthromyalgical effects, and only the non-supervised context was associated with adverse general symptoms. Finally, having a history of alcohol use disorder was negatively related with adverse arthromyalgical events. However, in a previous piece reporting on this same research, we described the apparent benefits of ayahuasca in reducing drug and alcohol consumption [17], so experiencing adverse physical effects does not seem to be related to ayahuasca’s efficacy in improving substance use disorder. This same survey also found positive results in anxiety, depression and general mental health and wellbeing [5, 12].

### Mental adverse effects

The frequency of adverse mental health effects was relatively high (55.4%), with a similar frequency being found for the emotional-cognitive and altered perception factors (42% and 38.3%, respectively; see preliminary analyses). Although the frequency of any adverse mental health effects was high, only “hearing or seeing things that other people do not hear or see” was observed in 28.5% of the ayahuasca users. In relation to this response and also “visual distortions” it is important to note that while we have assumed this to be an adverse experience, some respondents reporting these changes specifically mentioned in subsequent qualitative responses that they had considered these positive not adverse effects. Caution is therefore advised in interpreting results related to these two items. Other mental health adverse effects were reported in less than 21.0% (“feeling disconnected or alone”) of the participants. However, there was a low frequency of severe adverse effects (4.4% was the highest frequency, for “visual distortions”). Moreover, 11.9% of the participants reportedly needed professional mental health support for the adverse effects they experienced. For most participants reporting adverse mental health effects, the duration was identified as being for less than a week. While previous research has found long-lasting, severe adverse mental health effects in rare cases [53], other research has found that especially challenging adverse effects may improve psychiatric conditions [50], while other work from this study has reported the number of adverse mental health effects reported to be negatively associated with current mental health and perceived improvement in psychological wellbeing [5]. Other research has also found that adverse effects can be important enough to interfere in individual daily lives [52]. So, future studies should focus on follow-up and the evolution of the adverse effects when they appear.

History of ayahuasca use, age at initial ayahuasca use and lifetime use were not related with the adverse mental health effects. Regarding the clinical variables, only a previous diagnosis of anxiety disorder increased the likelihood of adverse emotional-cognitive effects, and previous physical health conditions increased the likelihood of altered perception adverse effects. All non-religious contexts (traditional, non-traditional and non-supervised) increased the likelihood of emotional-cognitive and altered perception adverse effects. Adverse emotional-cognitive and altered perception effects were more likely to be observed in females, younger participants, and those unmarried.

Adverse mental health effects were not significantly associated with ayahuasca history of use variables. This finding, while intriguing, is in accordance with the early age of ayahuasca initiation in traditional contexts, both Indigenous and in ayahuasca churches, where researchers failed to find long-term neuropsychiatric alterations [64, 65, 67, 68]. Over the last decade ayahuasca has increasingly been perceived to have therapeutic effects, leading to an increasing number of people accessing ceremonies as a complementary medicine or self-care practice. This situation has resulted in rare physical and/or mental health issues being more commonly seen in non-traditional contexts [10, 11].

Although this is the first study with a large sample to analyse ayahuasca’s adverse effects, some limitations must be noted. The study’s retrospective design and the fact that data were collected online make it impossible to know the degree of accuracy of the answers, and the sample is impacted by a self-selection bias. However, the large sample size makes this study, up until now, the most important source of information regarding ayahuasca’s adverse effects. Bias is commonly reflected in these kinds of studies in answers regarding positive effects, but this seems not to be the case in this study based on the high prevalence of adverse effects reported. In fact, most participants reported some adverse effects. Another limitation is the unknown combination of the plant materials used in ayahuasca brews consumed by the sample, meaning that it is not possible to consider the impact of this variable on adverse effects. This should be done in future naturalistic and clinical studies. Finally, although the sample included participants from more than 20 countries, the participants from Latin American countries, including Brazil especially, were over-represented, which could affect the results. However, the sample could also be considered a real representation of ayahuasca users’ global distribution.

Some conclusions should be highlighted from the study results. It may be significant that despite ayahuasca possibly inducing adverse physical and mental health effects, some of them potentially severe, as is the case of the neurological effects, users generally tolerate them well and continue using ayahuasca. This may be because using ayahuasca in group and ceremonial settings, especially in a religious context, seemingly protects users from developing more adverse side effects. This study found a relationship between using ayahuasca in non-religious settings and experiencing adverse effects, however, this could be due to the characteristics of users in each context. On the other hand, it is interesting that while patients with some psychiatric disorders experienced more adverse effects, having other psychiatric disorders reduced the likelihood of experiencing adverse effects. In fact, in a different analysis of other variables using this same sample, we found improvements in anxiety and depression [12], mental health and wellbeing [5] and in the use of drugs and alcohol [17]. Thus, most of the adverse effects reported here may be considered normal effects of ayahuasca use, and the relationship between experiencing adverse effects and the improvement of psychiatric disorders should be further studied.

Future studies should ask about the severity of both physical and mental effects, as well as about their duration. Moreover, in this study using a logistic regression analysis, a complex pattern of relationships between the study variables and the ayahuasca adverse effects is suggested. Thus, the study of the indirect effects and mediation structures between the studied variables could help us to improve our knowledge about the ayahuasca adverse effects and their relationships with context variables. Finally, it will be interesting to test how the studied variables are related to the improvement or aggravation of psychiatric disorders in the short or long-term.

Our results have very important implications in terms of public health relating to a traditional practice that has expanded internationally, and which is increasingly sought out by large numbers of non-native users in traditional locations. Ayahuasca has notable, although rarely severe, adverse effects according to the standards used for assessing prescription medicines. In that sense, ayahuasca practices can hardly be assessed with the same parameters used for prescription medicines, since the myriad of its effects include challenging experiences that are intrinsic to the experience, some of which are considered as part of its healing process. Like in most of psychotherapeutic processes, the therapeutic benefit may arise after having passed through various difficult processes. Shanon (2004) [69], describes this as follows:

*“One aspect of the Ayahuasca experience is a profound self-analysis*. *One is cruelly confronted with one’s self and one finds oneself having no other option but to address issues that are often neither easy nor pleasant to handle*.”

A participant in our study also described this process:

*“I have had numerous experiences where ayahuasca has brought difficult patterns into my awareness in my daily life*, *which is never comfortable but always results in growth in the end*.”.

In the context of such experiences, it is not surprising that a process of psychological integration and assimilation may be required. Furthermore, ayahuasca is not considered just a psychotherapeutic practice, but also a spiritual one. According to some spiritual traditions, the gaining of such spiritual insight will also involve initiates facing certain challenges.

The most worrisome effect is the possibility of induced psychiatric conditions that may persist if not properly treated [53]. However, this has previously been estimated to occur less frequently than in the general population [48]. Compared to other substances, persistent drug-induced mental health problems such as hallucinogen-persisting perception disorders (HPPD) are rarely reported among ayahuasca users [70]. However, a history of past trauma can put vulnerable patients at risk if they use mind-altering substances without proper guidance or psychological / spiritual support [71]. In that sense, as we have found in the current study, non-supervised contexts may increase the probability of suffering an adverse even. Another known health risk is the potential for drug interactions with some of the MAOI components of the brew. Concomitant use of certain prescription drugs (especially serotonergic substances) can increase adverse health effects, including the risk of serotonin syndrome [48]. On the other hand, it has been impossible to directly relate a single death to ayahuasca use [72]. There is also no evidence that ayahuasca has substantial or persistent abuse potential [18, 48]. Learning more about which conditions may be related to the occurrence of adverse effects of ayahuasca could help ayahuasca providers and guides to better screen users before administration and to provide better targeted integration support after consumption.

In sum, the international expansion of ayahuasca practice creates a series of new challenges for global public health policy and regulation. These include: 1) the intrinsic cultural complexity of making policy decisions in the absence (typically) of representatives with traditional knowledge; 2) the consideration of such healing practices based on the standards of biomedical medicine and its practices, where safety and efficacy do not necessarily refer to the same physical and psychological processes; and, 3) the possible recognition of such practices as therapeutic tools for self-care and mental health treatment in a globalized world, where instead of exporting medical systems cultures are importing them. The case of ayahuasca seems a paradigmatic example of how Western countries are trying to incorporate medical knowledge from other cultures into their informal self-healing practices [8]. It will be necessary to approach all these challenges will with open dialogue between different academic perspectives and epistemologies where traditional knowledge can dialogue with the scientific community.

## Supporting information

S1 File (DOCX)
