# Supplementary material for: Adverse effects of ayahuasca: Results from the Global Ayahuasca Survey
Source: PLOS Glob Public Health. 2022 Nov 16;2(11):e0000438. doi: 10.1371/journal.pgph.0000438 (PMC10021266; doi:10.1371/journal.pgph.0000438)
Supplement: S1 File — (DOCX) [file pgph.0000438.s001.docx]

| **Table A: Adverse physical effect components variance^1^** | | | |
| --- | --- | --- | --- |
| **Components** | **Eigenvalue** | **Variance** | **Acumulated variance** |
| 1 | 2.14 | 21.40 | 21.40 |
| 2 | 1.05 | 10.48 | 31.88 |
| 3 | 1.05 | 10.46 | 42.35 |
| 4 | 0.94 |  |  |
| 5 | 0.91 |  |  |
| 6 | 0.86 |  |  |
| 7 | 0.84 |  |  |
| 8 | 0.79 |  |  |
| 9 | 0.72 |  |  |
| 10 | 0.71 |  |  |
| ^1^ Principals component analysis. | | | |

| **Table B: Adverse physical effects factors loading^1^** | | | |
| --- | --- | --- | --- |
| **Adverse Effects** | **Generalsymptoms** | **Arthromyalgia** | **Neurological** |
| Abdominal pain | 0.63 |  |  |
| Vomiting/nausea | 0.62 | -0.21 | 0.18 |
| Breathing difficulties | 0.53 | 0.15 | 0.14 |
| Chest pains | 0.52 | 0.24 |  |
| Headache | 0.48 | 0.25 |  |
| Stiff/swollen joints |  | 0.74 | 0.16 |
| Aching muscles | 0.20 | 0.69 |  |
| Coughing/wheezing | 0.28 | 0.40 |  |
| Fits or seizures |  | 0.16 | 0.74 |
| Fainting | 0.17 |  | 0.70 |
| ^1^ Varimax rotation procedure. | | | |

| **Table C: Adverse mental health effect components variance^1^** | | | |
| --- | --- | --- | --- |
| **Components** | **Eigenvalue** | **Variance** | **Acumulated variance** |
| 1 | 4.45 | 44.54 | 44.54 |
| 2 | 1.43 | 14.26 | 58.80 |
| 3 | .75 |  |  |
| 4 | .70 |  |  |
| 5 | .64 |  |  |
| 6 | .50 |  |  |
| 7 | .46 |  |  |
| 8 | .44 |  |  |
| 9 | .33 |  |  |
| 10 | .30 |  |  |
| ^1^ Maximum likelihood procedure | | | |

| **Table D: Adverse mental health effects factors loading^1^** | | |
| --- | --- | --- |
| **Adverse Effects** | **Emotional-cognitive** | **Psychotomimetic** |
| Feeling nervous, anxious, or on edge | .73 | .33 |
| Not being able to stop or control worrying | .72 | .33 |
| Little interest or pleasure in doing things | .67 | .26 |
| Feeling down, depressed, or hopeless | .80 | .25 |
| Feeling disconnected or alone | .75 | .31 |
| Difficulty knowing what is real and not real | .54 | .44 |
| Nightmares, or disturbing thoughts, feelings, or sensations | .66 | .45 |
| Hearing or seeing things that other people do not hear or see | .30 | .78 |
| Visual distortions | .24 | .64 |
| Feeling “energetically attacked” or a harmful connection with a “spirit world” | .48 | .51 |
| ^1^: Rotation procedure Promax. | | |

| **Table E: Between context of ayahuasca use differences in the independent variables studied^1^.** | | | | | | | | | | |
| --- | --- | --- | --- | --- | --- | --- | --- | --- | --- | --- |
| Variables | Religious  (3,774) | | Traditional shaman (1,656) | | Non-traditional supervised (2,090) | | Non-supervised (442) | |  |  |
|  | Mean | SD | Mean | SD | Mean | SD | Mean | SD | F_(d.f.)_ | *p* |
| Doses/year^2^ | 2.86 | 1.28 | .78 | 1.26 | .83 | 1.24 | 1.04 | 1.55 | 1,499.62_(3; 7,212)_ | <.001 |
| Lifetieme uses^2^ | 5.19 | 1.75 | 2.02 | 1.43 | 2.06 | 1.53 | 2.61 | 1.87 | 2,184.68_(3; 7,307)_ | <.001 |
| Last years uses^2^ | 3.29 | .93 | 1.38 | 1.03 | 1.38 | 1.09 | 1.68 | 1.25 | 1,769.55_(3; 6,114)_ | <.001 |
| SIMO score | 69.29 | 15.08 | 67.54 | 19.57 | 69.24 | 18.16 | 69.73 | 19.08 | 4.46_(3; 7,579)_ | .004 |
| Age | 40.72 | 12.88 | 41.55 | 11.32 | 40.35 | 11.45 | 39.66 | 12.97 | 4.07_(3; 7,551)_ | .007 |
| Age of onset | 26.22 | 12.86 | 36.24 | 10.74 | 35.23 | 10.80 | 32.27 | 10.80 | 386.88_(3; 7,596)_ | <.001 |
| Nº phsysical conditions^3^ | .27 | .58 | .34 | .66 | .31 | .62 | .32 | .66 | 16.47_(3)_ | .001 |
|  | n | % | n | % | n | % | n | % | χ^2^_(3)_ | *p* |
| Female | 1,711 | 47.1 | 790 | 50.4 | 962 | 48.3 | 135 | 33.1 | 39.83 | <.001 |
| Anxiety disorder | 392 | 11.4 | 233 | 15.9 | 322 | 17.3 | 77 | 20.1 | 51.57 | <.001 |
| Depressive disorder | 505 | 14.6 | 340 | 23.9 | 481 | 25.8 | 99 | 25.8 | 119.66 | <.001 |
| Substance use disorder | 343 | 9.9 | 127 | 8.7 | 186 | 10.0 | 70 | 18.3 | 31.42 | <.001 |
| Alcohol use disorder | 385 | 11.1 | 129 | 8.8 | 184 | 9.9 | 37 | 9.7 | 6.69 | .08 |
| ^1^: ”(n)” indicates valid (non-missing) sample for each item; ^2^: variables presiously Ln transformed, see Statistical Analysis section. ^3^: Kruskal-Wallis test and reported mean and S.D. for the variable | | | | | | | | | | |
